# Global trends of local ecological knowledge and future implications

**DOI:** 10.1371/journal.pone.0195440

**Published:** 2018-04-05

**Authors:** Shankar Aswani, Anne Lemahieu, Warwick H. H. Sauer

**Affiliations:** 1 Department of Ichthyology and Fisheries Sciences, Rhodes University, Grahamstown, South Africa; 2 Department of Anthropology, Rhodes University, Grahamstown, South Africa; 3 UMR Espace-Dev 228, IRD, Reunion Island, France; Michigan State University, UNITED STATES

## Abstract

Local and indigenous knowledge is being transformed globally, particularly being eroded when pertaining to ecology. In many parts of the world, rural and indigenous communities are facing tremendous cultural, economic and environmental changes, which contribute to weaken their local knowledge base. In the face of profound and ongoing environmental changes, both cultural and biological diversity are likely to be severely impacted as well as local resilience capacities from this loss. In this global literature review, we analyse the drivers of various types of local and indigenous ecological knowledge transformation and assess the directionality of the reported change. Results of this analysis show a global impoverishment of local and indigenous knowledge with 77% of papers reporting the loss of knowledge driven by globalization, modernization, and market integration. The recording of this loss, however, is not symmetrical, with losses being recorded more strongly in medicinal and ethnobotanical knowledge. Persistence of knowledge (15% of the studies) occurred in studies where traditional practices were being maintained consiously and where hybrid knowledge was being produced as a resut of certain types of incentives created by economic development. This review provides some insights into local and indigenous ecological knowledge change, its causes and implications, and recommends venues for the development of replicable and comparative research. The larger implication of these results is that because of the interconnection between cultural and biological diversity, the loss of local and indigenous knowledge is likely to critically threaten effective conservation of biodiversity, particularly in community-based conservation local efforts.

## Introduction

The loss of biodiversity is currently increasing at an alarming rate globally [1,2]. Likewise, local and indigenous knowledge, particularly pertaining to plants and animals, is tailgating this loss. Local and indigenous ecological knowledge are understandings, beliefs, and practices that human societies develop longitudinally in relationship with their natural environment, and which are dynamic and co-evolving with social and ecological changes [3–5]. Since the 1990s there has been increasing awareness of the profound and ongoing social and economic transformation of rural and indigenous peoples [6,7]. This has led to a growing awareness of the fragile and eroding status of local knowledge systems [6,8,9], resulting in the publication of numerous studies reporting negative trends in indigenous knowledge loss [10]. The implications of this demise are likely to have grave repercussions as both biological and cultural (biocultural) diversity are interconnected [11–13] and are fundamental for both ecosystem health and human adaptive resilience to stochastic and anthropogenic driven environmental change.

The value of local ecological knowledge (LEK) (also referred to as indigenous and traditional ecological knowledge [IEK or TEK]) to mainstream conservation [11], began in the early 1990s when various researchers showed the cost-effectiveness and fairness to indigenous peoples territorial rights of integrating LEK into development and conservation projects [14]. While people can generate new and hybrid knowledge in everyday practice and interaction with the environment [15,16], the overall trend indicates that globalization processes in tandem with the standardization of educational systems are changing and impoverishing inter-generational cumulative environmental knowledge at a considerable rate [4,9,17,18]. The associated ongoing profound change of indigenous groups is dovetailing this rate of loss [6,7]. The processes that sustain or weaken peoples capacity to ‘adapt and regenerate’ local knowledge in the face of cultural change, acculturation, modernization, economic development and other transformative processes are not well understood [18]. Today, research on LEK is aiming to understand complex relations and causalities between the different components of LEK systems and their changes, and between LEK, policy and management, than to describe those knowledge systems per se (e.g. [7–9]). Despite the popular notion that local and indigenous knowledge systems are disappearing [19], the academic vision of LEK has progressively shifted from viewing LEK as a static body of knowledge to one of a dynamism. Knowledge is being hybridized through the accommodation of new forms of information or its exposition to external socio-economic drivers [20]. The causes of these changes are numerous and often addressed by correlating LEK variability and social drivers of change [4], and rates of change can be inferred when sets of data for two periods are available for the same study site (cross-sectional study). As such studies are rather scarce [21–24] authors generally resort to indirect measurement of change and allude to shifting baselines [25]. Authors assess differences in knowledge across generations of a same community [22], biodiversity loss and species misidentification [26], and acculturation [4] and markets integration effects [27]. The observed increasing cognitive dissonance between local and imported belief systems can typically result in an intergenerational loss in local people’s capacity to classify their environment correctly, manage their terrestrial and marine resources, and understand spatiotemporal changes locally [28–31]. These processes undermine overall livelihood resilience and make people, particularly in rural contexts, increasingly vulnerable to socioeconomic and environmental changes pushing them to further degrade their environment; thus the concomitant loss of biodiversity. It is important to note, however, that there are cases in which local people resist globalization and intrusion from outsiders, and organize themselves to defend their resources and territories [12, 14].

In this global review, we analyse the drivers of various types of local and indigenous ecological knowledge transformation and assess the directionality of the reported change. To date, no holistic study exists that reviews global trends in LEK. Hanazaki et al. (2013) [32] conducted a literature review of 84 studies but focused only on the loss of ethnobotanical knowledge. Here, we aim to expand and calibrate research on trends in local ecological knowledge change in its broadest sense. Generally, LEK is site specific knowledge held by a group of people about the various components of their environment and that may integrate both scientific and practical knowledge [33]. Traditional ecological knowledge (TEK) is similar but takes on a different definition in that it includes historical and cultural (systems of beliefs) dimensions [34]. In this study, we consider LEK in its most inclusive definition of ecological knowledge [35] including local, indigenous, traditional, and rural and which may be held by an indigenous community and/or local/rural communities from developing or industrialized nations. We also discuss the results in light of implications to resource management and conservation globally.

## Methods

**Fig 1:**
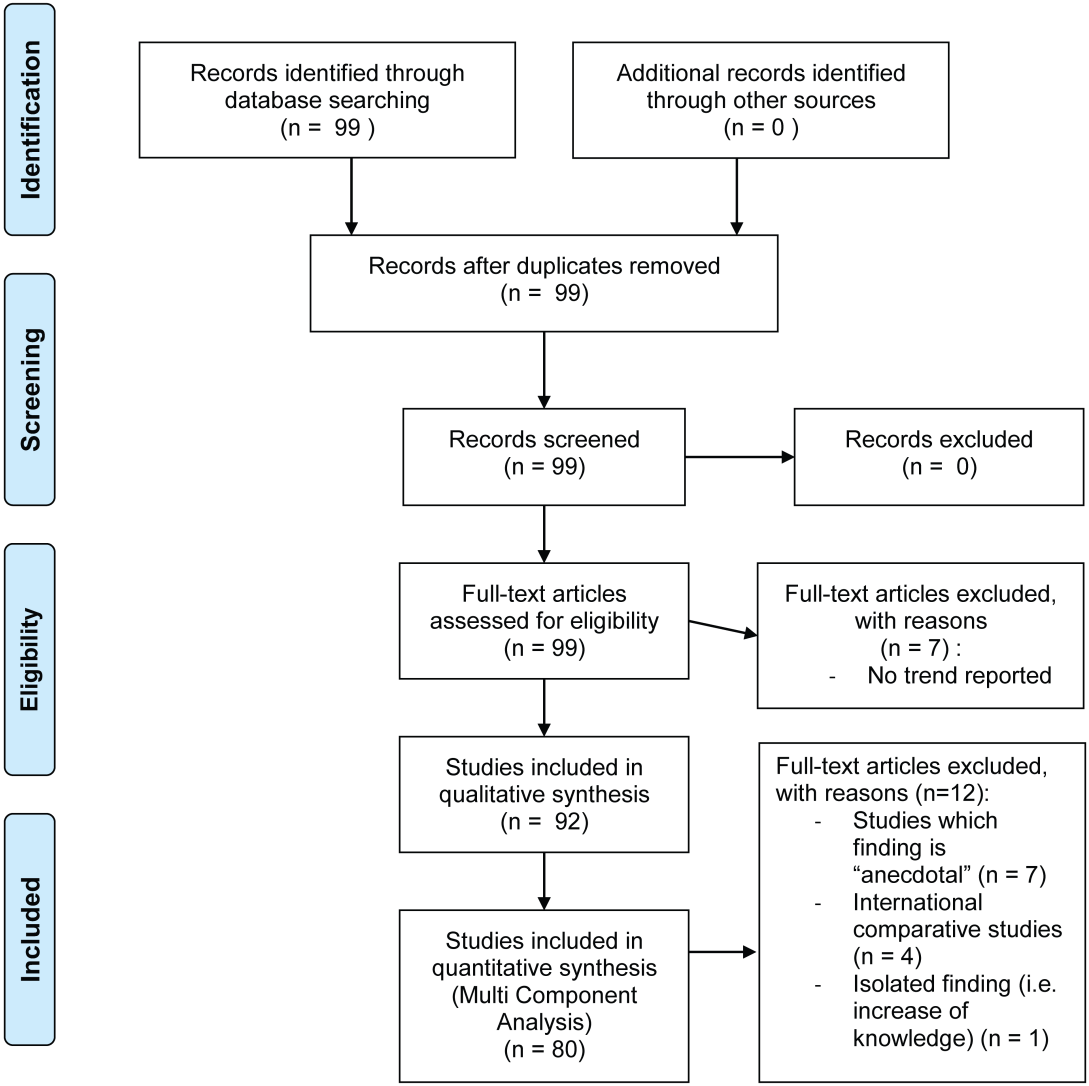
PRISMA flowchart describing the included/excluded literature.

An initial review was conducted using Google Scholar and Scopus and a range of keywords listed in Table 1 were used. During this process, additional keywords noting LEK loss, persistence, change or increase were added and all studies reporting any qualitative or quantitative LEK trends were included. Studies’ findings were categorised into 3 classes: (1) loss of LEK, when the knowledge is undergoing an eroding process across generations; (2) persistence and change of LEK, when the local knowledge is shown to be maintained over generations and/or undergoing some changes such as the creation of a new body of knowledge or its hybridization, and finally (3) ambiguous LEK status, when the findings were inconclusive and showing ambivalent dynamics or being unclear. An additional level of classification was applied to the analysis through categorising studies as: (1) evidence-based, if the finding was reported by respondents, clearly supported by data analysis, and trends referred to by the authors (2) non-evidence based, if findings were not supported by evidence yet reported by respondents and vaguely suggested by the authors, and (3) anecdotal, where a trend was not reported by respondents but suggested by the authors but could not be linked directly to the data analysis presented. When several studies were conducted by the same authors at the same location, we ensured not to record the sample twice (Fig 1).

**Table 1:** Words and association of words chosen for the research.

Root keywords	Additional keywords
Local, Traditional, Ecological, Knowledge, Ecoliteracy, Acculturation,	Loss, Decline, Shift, Change, Erosion, Shortage Acculturation, Dilution, Shifting baseline
	Maintenance, Persistence, Pockets (of knowledge)
	Increase, expansion

We considered descriptive variables contained in the papers including the kind of ecological knowledge examined (Topic), gender (Gender), and the country of study that we aggregated per regions (Region). Under the variable Topic, we distinguished medicinal knowledge (studies addressing plant knowledge exclusively for medicinal purpose), ethnobotanical knowledge (any other plant knowledge regardless to their use), agricultural and farming knowledge, animal-related knowledge, general environmental knowledge (e.g., knowledge about plant use for building and crafts, or about ecosystem functions and interconnectivity) and marine knowledge. The information about the drivers that were shown to be involved in LEK change was extracted from the studies and classified into 8 types of drivers. These include the existence of a generational shift with elders being more knowledgeable (Age), globalization processes including homogenizing acculturation (Globalization), modernization processes including technology introduction, urbanisation or modern health services (Modernization), the western formal education system (Education), market integration (Market), transmission pattern disruption (Transmission), various endogenous factors including demographic shift or beliefs and taboos erosion (Endogenous), and climate-related and environmental changes (Climate). Then, we considered the country where the studies were carried out, aggregated by regions (Region). While we would have wanted to integrate the year of study as a variable, the incompleteness in the information across the studies didn’t allow us to do this and we had to consider the decade of publication instead (Period). All the variables were categorized into categorical or binary variables (S1 and S2 Tables).

A Multi Component Analysis (MCA) was performed (using R [Version 3.3.3]) to examine relationships among the various variables extracted from the manuscripts. Used on categorical but non ordered data, MCA does not impose constraints on the data [36] as does a Principal Component Analysis (PCA). From the papers reporting a change in LEK, we excluded for the purpose of analysis the papers (1) which findings on LEK were classified as anecdotal, (2) were multi-nation studies under the category Region, and (3) the studies addressing more than one topic, hence making the classification difficult. All the variables used are listed in S1 and S2 Tables. The choice was made to include the primary data contained in the papers (e.g. Topic, Gender, various drivers, etc) into our analysis whereas the secondary data that we extracted from the papers (e.g. Period, Region) were used as supplementary variables. whereby they don’t contribute to the formation of the MCA space but are overlaid to display correspondences. To test dependencies between variables, chi-square statistical tests were performed between pairs of variables (tested at the significance level of 0.05) and redundant correlated variables were excluded from the analysis. Finally, a clustering was performed using the Hierarchical Clustering on Principal Component function (HCPC) of FactoMiner package and using the Ward classification method.

## Results

**Fig 2:**
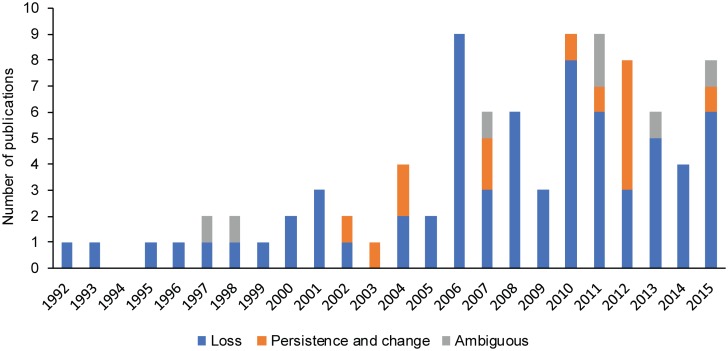
Temporal distribution of the 92 papers (year of publication) and LEK trend outcome.

First, a total of 92 papers emerged from our literature review research alluding to changes of any sort of LEK (S3 Table). Papers that we used were published between 1992 and 2016 and most of papers we identified were recent, i.e. published after the 2000s, with a peak in 2006, 2010 and 2011 (9 papers each) (Fig 2). The period of reported changes ranged from 1950 to 2013 and only 5 studies were found to use diachronic data [27,37–40]. A wide geographical representativeness was reflected with 50 countries represented. Brazil was the most studied country with 8 papers, followed by India and Bolivia, both with 5 papers. Regionally speaking, South-America was the most represented (25%) followed by Africa (20%), Asia (20%), and Europe (13%). Most studies addressed general ethnobotanical knowledge (65%) followed by agricultural and farming knowledge (11%), animal-related knowledge (11%), and marine knowledge and environmental knowledge (both 6.5%).

The loss of LEK was the predominant reported change (77%) followed by LEK persistence and transformation without loss (14%) (Fig 2). Studies’ finding with an “ambiguous” statement regarding the directionality of LEK trend accounted for 7.6%. For almost half of the studies, the trend was explained by a generational knowledge gap, with elders being more knowledgeable (45%). Market integration was an explanatory factor in 36% of studies, followed by modernization (40%), globalization (29%), and formal western education (32%). The gender differentiated knowledge was addressed in 75% of the studies. Among the studies that considered gender, 33% found an absence of gender dissociation in knowledge. Twenty three percent of the studies, mainly conducted on the American continent, found women to be more knowledgeable compared to men. Finally, 19% of the studies found men to be more knowledgeable especially in Asia and Europe. Over 50% of studies recommended some measure to protect LEK and around 33% of the publications gave some insights of biodiversity implications of changes in LEK. Finally, only 5 diachronic studies were identified, i.e., analysing two datasets for the same location at two different periods.

**Fig 3:**
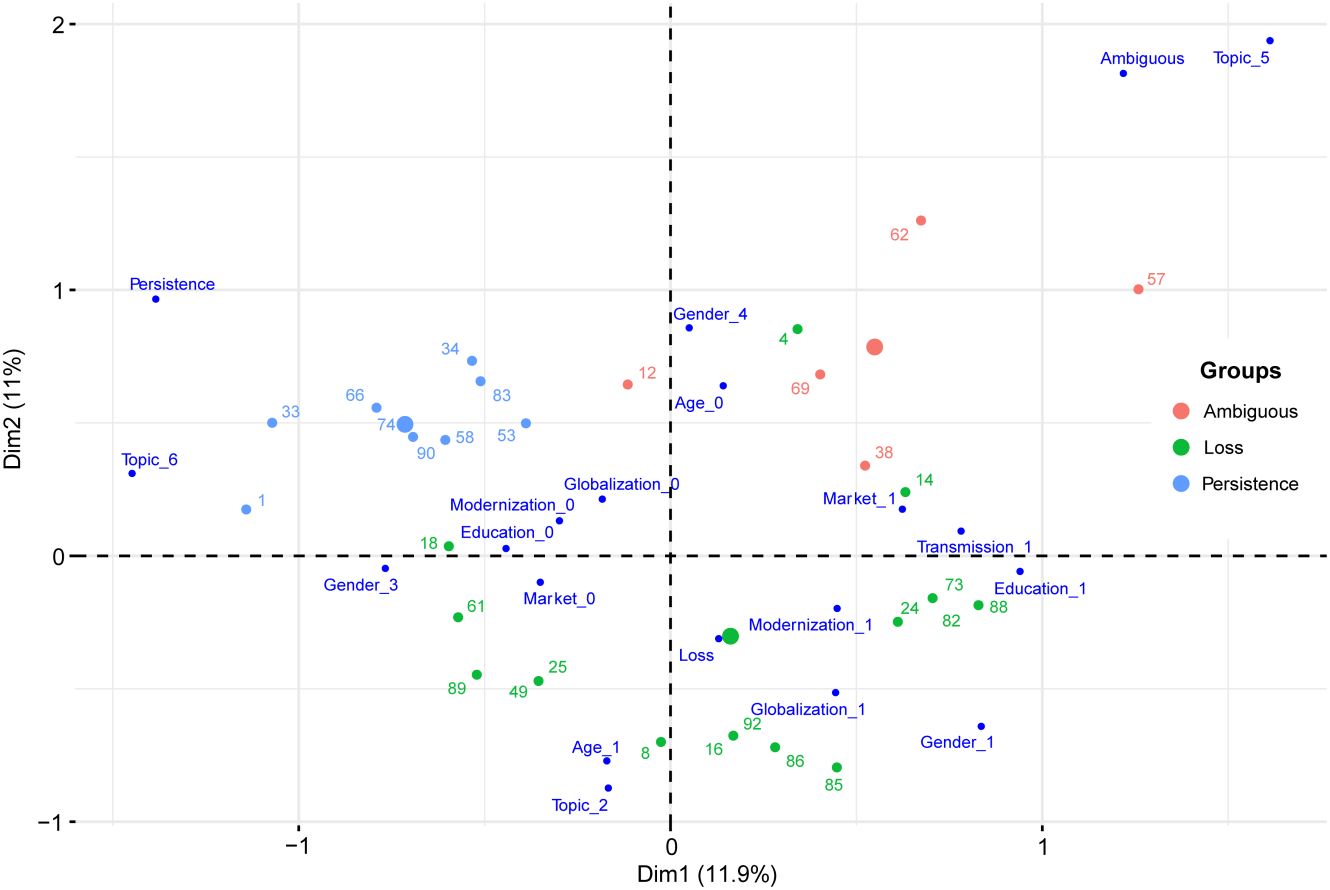
Factor map with the 20 modalities and 30 individuals having the highest contributions to the MCA axis.

The MCA was performed on a sample of 75 studies accordingly to the suitability selection criteria (S3 Table). The MCA was proven to be an efficient way of highlighting the relationships between the LEK status and the various drivers. While it could have added some value to the analysis, the time periods when the studies took place couldn’t be integrated to the analysis as this temporal information was missing for 24% of the papers. Chi-square tests demonstrated that the variable Endogenous was redundantly correlated to others variables, including the variable Trend (χ2 _(2)_ = 21.09, p = <0.001), Modernization (χ2 _(1)_ = 7.74, p<0.01), and to a lesser extent Education (χ2 _(1)_ = 4.88, p<0.05) and Age (χ2 _(1)_ = 3.96, p<0.05), and it was therefore removed from the analysis. The loadings of the first 5 principal components explained 49.3% of the variation in the data. The first dimension retained 11.94% of the variation in the original data and dimension 2 accounted for 11.02% of the variance (Fig 3). The following subsections describe the results for the 3 first axes:

Axis 1 (λ = 0.20): LEK persistence and Sea knowledge vs. Crafts and skills knowledge and ambiguous LEK status. The variance of the Axis 1 is more or less evenly distributed among the modalities. The strongest contributions are made by the modalities “Gender_3” (both gender equally knowledgeable) and “Topic_6” (Sea knowledge) which contributes at 9.66% and 8.25%. To a lesser extent, the “Market_1” (presence of market), “Topic_5” (Crafts and skills) and “Trend_1” (Loss of knowledge) modalities contribute to the variance. The variable Education is the variable the most significantly correlated to the axis (r = 0.41, p<0.001). On the right of the axis, the most important modalities are Crafts and skills (r = 0.7, p<0.001) and Ambiguous LEK status (r = 0.55, p<0.001) while the left of the axis is described by the modalities Sea knowledge (r = -0.66, p<0.001) Persistence and change of LEK (r = -0.61, p<0.001) and Both gender knowledgeable (r = -0.4, p<0.001).

Axis 2 (λ = 0.17): Medicinal knowledge and Loss of knowledge vs. Crafts and skills and Ambiguous LEK status. The variables Age (r = 0.49, p<0.001), Topic (r = 0.5, p<0.001) and Trend (r = 0.42, p<0.001) are the most significantly correlated to the axis 2. The modalities that are contributing the most to the variance are “Topic_2” i.e. Medicinal knowledge (12.4%), “Age_1” (elders more knowledgeable/SBS) (14.4%) and “Trend_3” (Ambiguous) (11.7%). On the top of the axis, the most important modalities are Ambiguous LEK status (r = 0.42, p<0.001), Crafts and skills (r = 0.66, p<0.001) and No gender addressed (r = 0.38, p<0.001) in opposition to Medicinal knowledge (r = −0.55, p<0.001) and Loss of knowledge (r = −0.49, p<0.001) at the bottom of the axis.

Axis 3 (λ = 0.16): Agricultural and farming knowledge and modernization vs Crafts and skills. The most important contribution to the third axis is made by the variables Topic (r = 0.57, p<0.001) and Modernization (r = 0.34, p<0.001). The modalities contributing the most to the axis variance are “Topic_3” (Agricultural and farming knowledge) (23.6%) and “Modernization_1” (13.1%). On the top of the axis we find that the most important modality is Crafts and skills (r = 0.53, p<0.001) whereas the bottom of the axis is mostly described by the modalities Agricultural and farming knowledge (r = -0.7, p<0.001) and “Region_4” (Europe) (r = -0.45, p<0.001).

**Fig 4:**
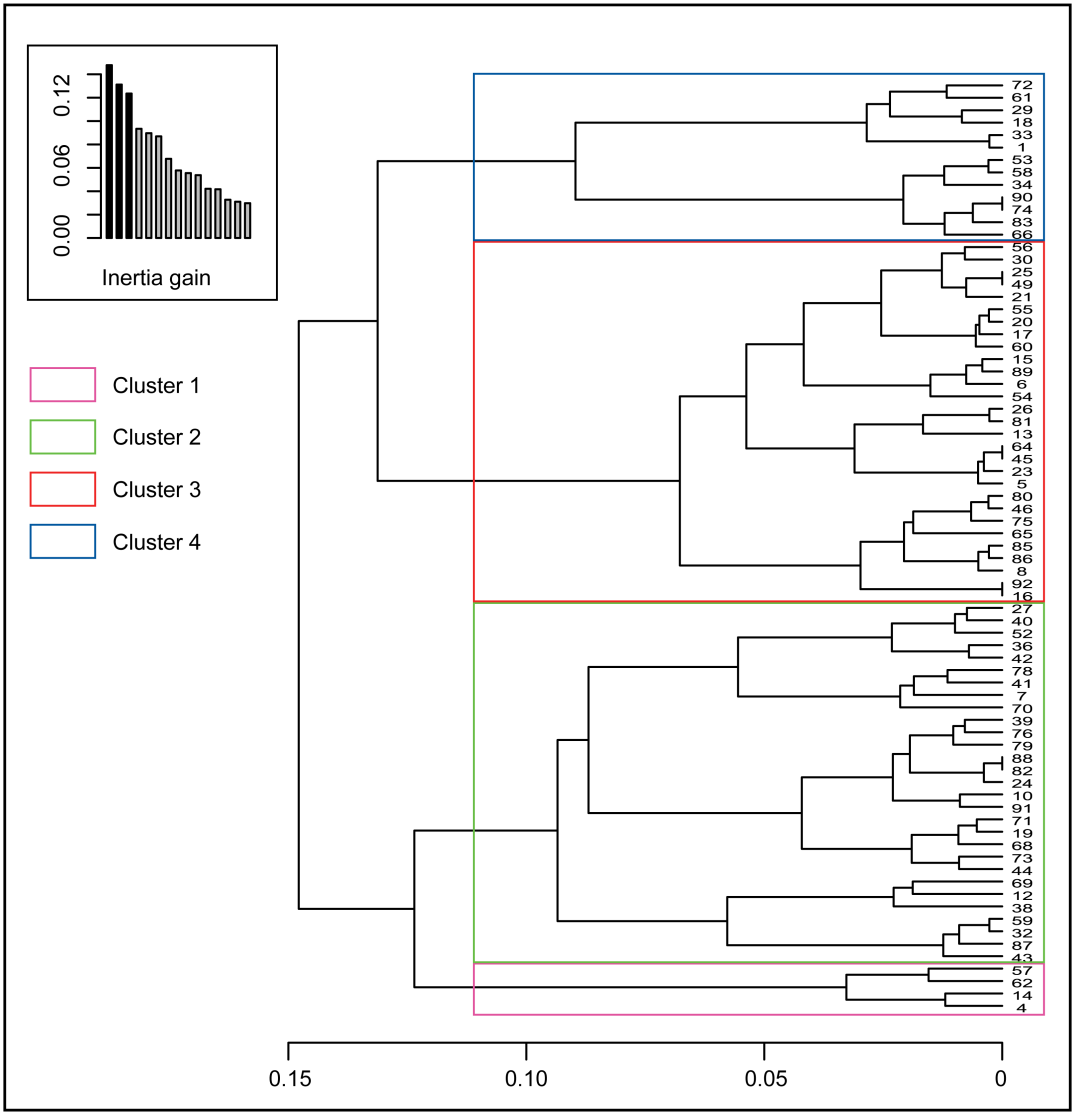
Classification tree and clustering in 4 clusters using the ward method (FactoMiner, R.3.3.3).

The clustering process produced 4 clusters (Fig 4): The first class (n = 13) contains studies which find a Persistence of LEK (90% of the papers finding a persistence are in this category). This persistence is generally attributed to the existence Endogenous factors (the maintenance of systems of taboos and beliefs) (study 58)[41], sedentary lifestyle (33), maintenance of traditions and transmission patterns (66, 74, 83), flexibility and adaption in techniques (34) or demographic shift (18). The studies contained in this class did not find a discernible difference between men and women in relation to LEK (1, 18, 33, 66, 74, 90) or didn’t address gender at all (34, 53, 58, 72, 83). The cluster is characterized by a slight predominance of studies carried out in Asia as they account for 46% of the studies in the cluster (29, 58, 61, 72, 74, 83), but only 37% of the modality subsample.

The second cluster (n = 29) is mostly made of studies dealing with Medicinal knowledge, almost exclusively reporting a Loss of knowledge (96% in the cluster). The driver Age describing a generational shift in knowledge, i.e., elders more knowledgeable is also well represented (86% in the cluster). Almost half of the papers (41%) contained in this cluster had their study site located in South-America (8, 13, 20, 21, 25, 30, 5, 54, 55, 80, 85, 86).

The third cluster (n = 29) is the one comprising the highest number of papers. It is characterized by a predominance of studies dealing with Ethnobotanical knowledge (55% in the cluster) and to a lesser extent Agricultural and farming knowledge (20% in the cluster but 75% of the subsample) and dramatically report a loss of knowledge (89%). In this cluster we find the predominance of the drivers Modernization (65%), Education (55%) and Market (55%). The region Europe is well represented with 73% of the subsample being found in this cluster (27% in the cluster) (32, 38, 38, 42, 43, 59, 70, 87). The fourth cluster which is the most homogeneous cluster but also the smaller one (n = 4) is only described by two variables. The totality of the papers that are dealing with the topic Crafts and skills are contained in this cluster (4, 14, 57, 62) which is, besides, exclusively made of papers dealing with this topic. Half of these papers found an Ambiguous LEK status (50% in the cluster) (57, 62).

The variables Trend and Topic (p<0.001), which explained most of the MCA axes variance, drove the clustering process. More specifically, the modality Crafts and Skills of the variable Topic (Topic_5) and the modality Ambiguous of the variable Trend (Trend_4) highly contributed to the three first axis and pooled together explain why they formed a consistent cluster (cluster 4). Because the variance of the 3 axes was mostly explained by these two modalities, it decreased the explanatory power of the analysis. Nevertheless, the clustering process showed a relative consistency in grouping the papers according to their finding and in bringing forward the principal drivers associated with each type of LEK status. The list of papers and corresponding cluster is found in the S3 Table.

## Discussion

The loss of knowledge is a general finding of this paper, albeit it should be noted that these studies do not find a uniform loss across all knowledge types. Ethnobotanical and medicinal knowledge were the knowledge types most studied. While studies focusing on South America were in the majority, a broad and geographically representative distribution was nevertheless present. The gendered difference in knowledge was examined in half of the studies. While a small majority of these studies found that LEK loss or change was equally present for both genders, it would appear that LEK changes are more likely to apply to women more than men. Some regions were associated with a gender-specific LEK trend, as in South America where LEK was shown to be held mainly by women, but with changes in LEK being observed mostly in relation to men. In Asia, conversely, LEK changes were reported to affect women more.

According to our findings, medicinal and ethnobotanical knowledge types were the types most significantly impacted upon by the loss of LEK. However, it should be borne in mind that studies focusing on these knowledge types constituted the biggest sample in our literature review. As such, this finding should be treated with some caution. Studies examining medicinal and ethnobotanical knowledge types also found that men were more likely than women to be affected by the loss in LEK. Although the two topics are not mutually exclusive, dissociating medicinal knowledge from ethnobotanical knowledge in our analysis proved to be a relevant choice. The clustering operation highlighted some differences in how the two topics relate to different drivers. The erosion of medicinal knowledge was highlighted by showing a difference in knowledge between younger and older generations, with younger generations being less knowledgeable. This difference in knowledge is either shown by comparing younger and elder generations’ level of knowledge, or from analysing diachronic data for the same generation. In some cases, authors identified a loss of transmission patterns as causing the generational shift [42–44].

Several exogenous factors are triggering major changes in societies and lifestyles, thereby interrupting the oral, inter-generational transmission of knowledge from elder generations to younger ones [45]. For ethnobotanical knowledge, the loss trend was mostly explained by homogenizing acculturation, various globalization processes, or the presence of formal Western education systems. The integration of tourism [46], economic shifts, from primary to secondary sectors [47,48] and the resulting rural exodus [46,49], urbanization processes [48–50], deforestation [51] and modern agricultural practices [47,51] were all shown to be deleterious to LEK. Formal Western education systems also contribute to weaken LEK, especially in relation to younger generations [47,49] by depriving children from daily interactions with plants [51] and elders. The fact that medicinal plants are less likely to be a part of a market, when compared to edible or logging plants, could explain why the fluctuation in relation to medicinal knowledge is less dependent on markets and accordingly less affected by these processes. In the developed world, persisting agricultural and agro-silvopastoral “pockets of knowledge” [20] were found to erode in the presence of modernization and industrialization processes [52–54], people migration [35], and a shift in energy production from fossil fuels to renewable energy [55,56]. The implementation of a common EU agricultural market and the lack of diversification of activities were also shown to be involved in this erosion process [52]. In those areas, the modern agricultural techniques induced a masculinization of tasks, thus causing a gender-specific specialization of knowledge to the benefit of males [5,53,54].

The persistence of knowledge found in some studies was generally related to endogenous processes such as the persistence of a system of beliefs [41], of the transmission patterns [57–59] and more generally to the maintenance traditional practices [40]. Given that LEK is dynamic [22], its exposure to external forces sometimes results in the creation of a hybrid knowledge [60]. Rather than a loss, Mathez-Stiefel et al. ([23], p.917) talk about “a process of adaptation to a changing context, and some authors believe that traditional knowledge is more likely to change or hybridize than to be lost” [61]. Indeed, variation in knowledge can happen through various processes that are not necessarily linked to knowledge erosion [62]. For instance, the capacity to adapt to perturbations can ensure local knowledge persistence and/or transformation. As shown by Hamlin and Salick’s study on Yanesha people in the upper Peruvian Amazon [39], the flexibility in the agricultural techniques (e.g., intercropping, crop rotation, etc.) have been ensuring the persistence of LEK through its adaptation. Aswani and Albert also pointed out in their study in the Solomon Islands the existence a new knowledge being generated as people adapt to environmental changes [37].

Therefore, in some cases, market integration processes result in the conservation of LEK, through the enhancing of local products or practices [63–65]. As shown by Guest et al. [64] in Ecuador, the integration of the market economy in shrimp fisheries, instead of causing complete knowledge erosion, resulted instead in the creation of local ecological knowledge through its contribution to the production of a new body of skills-related knowledge. Similarly, the maintenance of LEK in relation to medicinal plants in the Tanga region, northeastern Tanzania, was shown to be supported as a result of the plants having a value in the regional medicinal market system [65]. This transformation and hybridization of knowledge explains why a lot of these studies are inconclusive in relation to whether or not external forces negatively impact LEK. On the one hand, theoretical knowledge is often found to be eroding, but on the other hand skills knowledge is being maintained and even increased by exogenous factors. In their study on the Tawakha Indians of Honduras, Godoy et al. ([63], p.229) conclude by saying that “markets may be associated in systematic ways with both the loss and the retention of indigenous knowledge”. A similar assessment was made by Reyes-García et al. ([66], p.376) in relation to the Tsimane’ people in Bolivia, where it was found “that some forms of economic development can take place without eroding local ecological knowledge”. Indeed, in these studies, market integration was found to either be erosive, as in the case of wage labour, or to positively contribute to the maintenance of LEK, for example through the sale of timber/non-timber forest goods or farm products. A subsequent study carried out by the same authors in the same region confirmed the initial finding of a loss of knowledge in relation to medicinal plants in contrast to the maintenance of knowledge of plants used for canoe building and firewood (skills) [67]. In sum, hybridization, in our experience, does indeed entail the loss of “old” or “traditional” knowledge, but its “mixing” with “foreign” ways of thinking and the inclusion of “new” ideas into the local or indigenous social economy and mind-set does not result in a net loss of the LEK repertoire.

Despite all these caveats, the overall trend is a loss of knowledge globally. Two studies analysing cross-sectional data found a rate of 1.9 and 2.2% annual loss of ecological knowledge. In their study on the knowledge of plant use among Tsimane’ people (Bolivia), V. Reyes-Garcia et al. [27] found an annual rate of 2.2% per year assuming a linear change from 2000 to 2009. Similarly, Aswani and Albert [37] in a study on local vernacular fish names in the Western Solomons, reported an annual loss of 1.9%, assuming a linear net loss of “traditional” names between 1995 and 2011 (new invented names were not accounted for). While these studies are only a small sample size and only looked into to the loss of organism’s names (and not taxonomic distinctiveness identification) and as such can’t be considered representative of a global rate of loss, they do hint at an ongoing trend of cultural knowledge erosion. It follows that if we take these studies as a reference point and assume an average linear change of 2% per annum, LEK systems as they exist today (which are already changed and hybridized), would be entirely transformed globally in 50 years (by 2066).

### LEK erosion implications

LEK systems as cultural diversity are deeply interconnected with biological diversity and the erosion of one can profoundly impact the other [68,69]. Where knowledge about plants is lost, there is a risk of a knock on effect leading to the erosion of natural resources [70], a loss of biodiversity [71,72] and the disappearance of plant species [47,59,73–75]. Conversely, the decline in the number of used species and their diversity can result in a loss or transformation of knowledge [26,76,77]. This ongoing erosion of LEK through cultural and linguistic extinction (e.g. [4,7,48,57,78,79]) has also been shown to undermine conservation efforts [80]. For instance, Gorenflo et al. [81] have shown that there is a geographical co-occurrence of linguistic and biological diversity in many parts of the world and that changes in one can affect the other. The loss of languages observed in Mexico among the Zapotec communities of the Oaxacan Isthmus [47] or among the communities of the Sierra de Manantlan Biosphere Reserve [8] is believed to have affected conservation efforts in these areas. This acculturation process, which started with colonisation in many cases [48], implies the importation of new lifestyles based on new communication technologies and western food [46,50,70]. Such a “nutrition transition” [48] has many secondary implications, including food insecurity and micronutrient deficiency [70,82] and a decrease in diet diversity [70] triggering diseases like diabetes [48]. These changes in lifestyle affect young people more, who tend to adopt new values to the detriment of traditional practices [8,49]. In places where globalization processes were not present and where traditions were conserved [23,35,40,41,51,57], the young were found to be responsible for the maintenance of traditional knowledge. Indeed, where cultural erosion occurs, a process of LEK devaluing is triggered, leading to a disinterest in younger generations in relation to traditional practices which are often perceived as primitive and “backward” [44,70,83,84]. One of the main reason for local knowledge loss is the low value attached to it [85]. This, in turn, sets the stage for a “desensitisation to ecosystem change” [86] and to concomitant negative conservation effects.

The preservation of LEK can be brought about through a range of measures that ensure the maintenance of transmission patterns [51] through contributing to LEK recognition and valuing. These measures include the implementation of educational programs that include traditional knowledge teaching programs at school [44,87–91], more government intervention through the reinforcement of institutions and laws [26,41,46,79,92], new ways of web-based LEK transmission, or the implementation of revitalization projects through the rehabilitation of old traditional practices [93,94]. Studies in Africa and South America, often recommend a focus on the improvement of local conservation methods that ensure communities autonomy and knowledge perpetuation. That is, alternative preservation techniques [70], ex situ and in situ biodiversity conservation [50,72,95], and extractive reserves [96]. In Austria, home gardening is likely to play a potential role in the propagation and the conservation of knowledge [97]. Finally, some findings show that local knowledge could be maintained through certain types of market integration [63–65], the development of ecotourism [98], the collaboration with industry agents on ecological labels [38], the emerging agro-ecological movements [54] and the creation of local cooperatives [99].

In sum, the current loss of LEK is problematic because millions of people still depend on wild and rural agricultural natural resources for their livelihoods. Indigenous communities are particularly vulnerable because they are the least developed, and have limited access to education and health services, have a short life expectancy, high infant mortality, and have high population growth rates (e.g. [100]). As their livelihood dependency on natural resources is high, natural resources are of critical cultural and economic importance. LEK (biological, climatic, etc.) helps people understand and take advantage of environmental unpredictability and variability, and to detect sudden environmental changes and adapt to them. Recent changes in climate across the world provide a further challenge as patterns of climate change are dynamic and highly heterogeneous across the Earth, so uniform responses across the globe to climate change should not be expected [100]. The implications of these changes to communities reliant on natural resources are highly complex and people’s capacity to detect environmental and climatic changes (or lack thereof) plays a key role in how they perceive risks associated with change. As the effects of climate change increase, the production of anticipatory and autonomous adaptation locally, which is affected by the perceived changes and causes of change, will become crucial for enhancing people’s well-being locally. Building socio-ecological resilience, therefore, will necessitate people’s capability to both learn swiftly and adapt to a rapidly changing planet. Hence, the loss of local knowledge can result in a diminished ability to cope with environmental alterations [32] and a decreased capacity to improve resilience locally.

### Future research

The quantitative assessment of LEK loss /change and rate of change is made difficult by the absence of cross-sectional data or “longitudinal data” [88] and scant research has been conducted to measure longitudinal LEK change [61,88]. Our literature review only identified 5 diachronic studies [27, 37–40]. When not identified from diachronic data, LEK changes are found from an observed difference of knowledge between youth and elders, a phenomenon defined by certain authors as “Shifting Baseline Syndrome”[25], or the fact that what a past generation understood as their natural environment is no longer recognizable and/or pertinent to a younger generation that are living in new environmental conditions (often in more degraded and resource poor environments). Nevertheless, many authors are cautious about making such assumptions and translating a simple correlation into a loss of knowledge [98]. These authors argue that the correlation often found between knowledge and age can be explained by an “age effect” [22], whereby elders have had a greater amount of time to accumulate knowledge [4,43,62,101,102]. Therefore, some argue that this correlation can be an “artefact” [89] that might imply a misleading impression of knowledge erosion. Looking at the present patterns of intracultural variability and connecting them with indicators of changing conditions (social variables, cultural or economic variables) has been another common way to indirectly assess the ongoing LEK trends [4,23]. Still, in order to accurately measure the velocity of LEK loss or change, diachronic assessments of local/traditional knowledge are required [21–24,103], but such studies are scarce. Future research on knowledge change needs more replicable comparative research on the transmission of ecological knowledge [40].

The integration of western science knowledge and local knowledge is one possible way to durably embed local/indigenous/traditional ecological knowledge and its related local population into decision making and natural resources management policies [64,104]. Even if the two sources of knowledge have common ground [105], LEK is more likely to detect extreme events and record historical changes, whereas western science adopts a synchronic temporality [106] (albeit it tends to be more effective at detecting protracted changes). It follows that these two knowledge systems can be complementary [105] and integrating both into resource management is often recommended [104,106]. The integration of local/indigenous and scientific knowledge also leads to an increase in sample sizes and time series, hence reducing uncertainties [104]. Additionally, LEK was in some cases found to be as accurate as and cheaper to produce/record than western knowledge [58,107]. It’s inclusion in management or development plans is also a necessary recognition of the basic cultural and territorial rights of local and indigenous people who are often subjected to environmental injustice.

The method of asking respondents about their perception of change through their lifetime should be utilised when longitudinal data are not available [88]. Finally, given the diverse drivers of LEK loss, research on environmental shifts and biodiversity loss drivers [26,31] will be central to future studies. This is important because the ongoing impoverishment of LEK is likely to impact biological diversity negatively [68,81]. This will further challenge conservation strategies in many part of the world where local and indigenous people closely interact with their environment.

## Conclusion

In a context where many conservation goals must be achieved, LEK can be a contributor to a multidisciplinary conservation approach as well as foster transdisciplinary approaches when locals become partners and collaborators. This is because it is a key component for a successful management system that can sustain local resources [108] and, therefore greater efforts are needed to develop methods to quantify LEK change [86], and LEK must be documented as much as possible before it is transformed or lost all together [73]. Further research on knowledge change in different local contexts is needed, as a lack of LEK research in developed countries may bias our perception and assumptions on so-called “residual” knowledge or “pockets of knowledge” which would be no longer relevant in a context of modernization. Just as the loss of biodiversity is impoverishing our world biologically, the loss of local and indigenous ecological knowledge is impoverishing our world socio-culturally. These two processes reciprocally enforce each other, leading to further negative consequences for conservation. Efforts, therefore, should be made not only to safeguard biodiversity but also indigenous peoples and their knowledge systems.

## Supporting information

S1 Table List of the binary variables used for the MCA (n = 75) with their description, the corresponding frequency and code displayed in the analysis. (DOCX)

S2 Table List of the categorical variables used for the MCA (n = 75) with their description, the corresponding frequency and code displayed in the analysis. (DOCX)

S3 Table List of the papers used in the MCA, with their ID, year of publication, country where the study was carried out, the topic addressed and their corresponding cluster number (Grey rows correspond to studies which were removed from the analysis after we applied the exclusion criteria). (DOCX)
