# Supplementary material for: Global trends of local ecological knowledge and future implications
Source: PLoS One. 2018 Apr 5;13(4):e0195440. doi: 10.1371/journal.pone.0195440 (PMC5886557; doi:10.1371/journal.pone.0195440)
Supplement: S1 Table — (DOCX) [file pone.0195440.s001.docx]

**Table S1.** List of the binary variables used for the MCA (n=75) with their description, the corresponding frequency and code displayed in the analysis.

| **Variable** | | **Description** | **Frequency** | **Code** |
| --- | --- | --- | --- | --- |
| **Binary variables** | Age | Generational knowledge gap with the elders more knowledgeable, Shifting Baseline Syndrome associated with loss | 0,45 | Age_1 |
|  |  |  |  | Age_0 |
|  | Market | Distance to a town, Wealth, Occupation | 0,36 | Market_1 |
|  |  |  |  | Market_0 |
|  | Modernization | Modern Health Services, Mechanization, Urbanization, Land dispossession (agriculture), Rural outmigration | 0,40 | Modernization_1 |
|  |  |  |  | Modernization_0 |
|  | Globalization | Acculturation, Lack of interest of the youth, Social networks, Language loss, Westernization, Religion, Tourism, (international) Migrations, Conservation programs, Western Science | 0,29 | Globalization_1 |
|  |  |  |  | Globalization_0 |
|  | Education | Western education, Educational programs | 0,32 | Education_1 |
|  |  |  |  | Education_0 |
|  | Climate | Climate changes, Droughts | 0,15 | Climate_1 |
|  |  |  |  | Climate_0 |
|  | Endogenous | Geographical location, Taboos and beliefs, Government or institution intervention (subventions), demographic shifts, Adaptation skills (flexibility in agriculture) | 0,20 | Endogenous_1 |
|  |  |  |  | Endogenous_0 |
|  | Transmission failure | Reported transmission patterns defects | 0,11 | Transmission_1 |
|  |  |  |  | Transmission_0 |
