# Supplementary material for: Global trends of local ecological knowledge and future implications
Source: PLoS One. 2018 Apr 5;13(4):e0195440. doi: 10.1371/journal.pone.0195440 (PMC5886557; doi:10.1371/journal.pone.0195440)
Supplement: S2 Table — (DOCX) [file pone.0195440.s002.docx]

**Table S2**. List of the categorical variables used for the MCA (n=75) with their description, the corresponding frequency and code displayed in the analysis.

| **Variable** | | **Description** | **Modality** | **Frequency** | **Code** |
| --- | --- | --- | --- | --- | --- |
| **Categorical** | Trend | The LEK trend finding in the papers | Loss | 0,80 | Trend_1 |
|  |  |  | Persistence and/or change | 0,13 | Trend_2 |
|  |  |  | Ambiguous | 0,07 | Trend_3 |
|  | Gender | The gender of knowledge | Females more knowledgeable | 0,23 | Gender_1 |
|  |  |  | Males more knowledgeable | 0,19 | Gender_2 |
|  |  |  | Both gender knowledgeable | 0,33 | Gender_3 |
|  |  |  | No gender addressed | 0,25 | Gender_4 |
|  | Topic | The type of LEK addressed in the study | Ethnobotanical knowledge | 0,33 | Topic_1 |
|  |  |  | Medicinal knowledge | 0,31 | Topic_2 |
|  |  |  | Animal knowledge | 0,11 | Topic_3 |
|  |  |  | Agriculture & farming | 0,12 | Topic_4 |
|  |  |  | Sea knowledge | 0,05 | Topic_5 |
|  |  |  | Crafts and skills | 0,08 | Topic_6 |
|  | Region | Data are aggregated by regions of the world | South-America | 0,27 | Region_1 |
|  |  |  | Africa | 0,17 | Region_2 |
|  |  |  | Asia | 0,21 | Region_3 |
|  |  |  | Europe | 0,15 | Region_4 |
|  |  |  | North-America | 0,12 | Region_5 |
|  |  |  | Oceania | 0,08 | Region_6 |
|  | Period of publication | Data are aggregated by decades | 1990s | 0,08 | Period_1 |
|  |  |  | 2000s | 0,44 | Period_2 |
|  |  |  | 2010s | 0,48 | Period_3 |
