# Supplementary material for: Global trends of local ecological knowledge and future implications
Source: PLoS One. 2018 Apr 5;13(4):e0195440. doi: 10.1371/journal.pone.0195440 (PMC5886557; doi:10.1371/journal.pone.0195440)
Supplement: S3 Table — (DOCX) [file pone.0195440.s003.docx]

**Table S3.** List of the papers used in the MCA, with their ID, year of publication, country where the study was carried out, the topic addressed and their corresponding cluster number (Grey rows correspond to studies which were removed from the analysis after we applied the exclusion criteria).

| **Paper ID** | **Author(s)** | **Year of publication** | **Country of the study** | **Topic addressed in the paper** | **Cluster** |
| --- | --- | --- | --- | --- | --- |
| 1 | Aswani and Albert | 2015 | Solomon Islands | Sea knowledge | 1 |
| 2 | Ahmed et al. | 2010 | China | Agricultural and farming knowledge | - |
| 3 | Ainsworth | 2011 | USA | Sea knowledge | - |
| 4 | Arunotai | 2006 | Thailand | Craft and land skills | 4 |
| 5 | Awas et al. | 2010 | Ethiopia | Ethnobotanical knowledge | 2 |
| 6 | Badshah and Hussain | 2010 | Pakistan | Medicinal plant knowledge | 2 |
| 7 | Barreau et al. | 2016 | Chile | Ethnobotanical knowledge | 3 |
| 8 | Begossi et al. | 2002 | Brazil | Medicinal plant knowledge | 2 |
| 9 | Benoit | 2008 | Burkina Faso | Medicinal plant knowledge | - |
| 10 | Benz et al. | 2000 | Mexico | Ethnobotanical knowledge | 3 |
| 11 | Biro et al. | 2014 | Romania-Hungary | Ethnobotanical knowledge | - |
| 12 | Bognounou et al. | 2011 | Burkina Faso | Ethnobotanical knowledge | 3 |
| 13 | Brandt et al. | 2013 | Bolivia | Agricultural and farming knowledge | 2 |
| 14 | Brosi et al. | 2007 | Micronesia | Craft and land skills | 4 |
| 15 | Byg and Balslev | 2001 | Madagascar | Ethnobotanical knowledge | 2 |
| 16 | Caniago and Siebert | 1998 | Indonesia | Medicinal plant knowledge | 2 |
| 17 | Case et al. | 2005 | Papua New Guinea | Medicinal plant knowledge | 2 |
| 18 | Crona | 2006 | Kenya | Sea knowledge | 1 |
| 19 | Cruz Garcia | 2006 | India | Ethnobotanical knowledge | 3 |
| 20 | de Albuquerque et al. | 2011 | Brazil | Medicinal plant knowledge | 2 |
| 21 | de Almeida et al. | 2010 | Brazil | Medicinal plant knowledge | 2 |
| 22 | De Beer and Van Wyk | 2011 | South Africa | Ethnobotanical knowledge | - |
| 23 | Della and Paraskeva-Hadjichambi | 2006 | Cyprus | Ethnobotanical knowledge | 2 |
| 24 | Dweba and Mearns | 2011 | South Africa | Ethnobotanical knowledge | 3 |
| 25 | Estomba et al. | 2006 | Chile | Medicinal plant knowledge | 2 |
| 26 | Etiendem et al. | 2011 | Cameroon | Animal knowledge | 2 |
| 27 | Fernandez-Llamazares et al. | 2015 | Bolivia | Animal knowledge | 3 |
| 28 | Flatie et al. | 2009 | Ethiopia | Medicinal plant knowledge | - |
| 29 | Gaillard et al. | 2008 | Indonesia | Sea knowledge | 1 |
| 30 | Gazzaneo and de Lucena | 2005 | Brazil | Medicinal plant knowledge | 2 |
| 31 | Godoy et al. | 1998 | Honduras | Animal knowledge | - |
| 32 | Gomez-Baggethun et al. | 2010 | Spain | Agricultural and farming knowledge | 3 |
| 33 | Guest | 2002 | Ecuador | Sea knowledge | 1 |
| 34 | Hamlin and Salick | 2003 | Peru | Agricultural and farming knowledge | 1 |
| 35 | Harousse et al. | 2012 | Morocco | Agricultural and farming knowledge | - |
| 36 | Horstman and Wightman | 2001 | Australia | Animal knowledge | 3 |
| 37 | Ianni et al. | 2015 | Italy | Ethnobotanical knowledge | - |
| 38 | Iniesta-Arandia et al. | 2015 | Spain | Agricultural and farming knowledge | 3 |
| 39 | Johann | 2007 | Austria | Ethnobotanical knowledge | 3 |
| 40 | Kai et al. | 2014 | China | Animal knowledge | 3 |
| 41 | Keller et al. | 2006 | Tanzania | Ethnobotanical knowledge | 3 |
| 42 | Kikvidze and Tevzadze | 2015 | Georgia | Animal knowledge | 3 |
| 43 | Kizos | 2013 | Greece | Agricultural and farming knowledge | 3 |
| 44 | Kodirekkala | 2015 | India | Ethnobotanical knowledge | 3 |
| 45 | Ladio and Lozada | 2004 | Argentina | Ethnobotanical knowledge | 2 |
| 46 | Lyon and Hardesty | 2012 | Madagascar | Medicinal plant knowledge | 2 |
| 47 | Mahonge et al. | 2006 | Tanzania | Medicinal plant knowledge | - |
| 48 | Maikhuri and Gangwar | 1993 | India | Ethnobotanical knowledge | - |
| 49 | Matavele and Habib | 2000 | Mozambique | Medicinal plant knowledge | 2 |
| 50 | Mathez-Stiefel et al. | 2012 | Bolivia/Peru | Medicinal plant knowledge | - |
| 51 | Maundu | 1992 | Kenya | Ethnobotanical knowledge | - |
| 52 | McCarter and Gavin | 2014 | Vanuatu | Animal knowledge | 3 |
| 53 | McMillen | 2012 | Tanzania | Medicinal plant knowledge | 1 |
| 54 | Meretika et al. | 2010 | Brazil | Medicinal plant knowledge | 2 |
| 55 | Monteiro et al. | 2006 | Brazil | Medicinal plant knowledge | 2 |
| 56 | Nolan and Robbins | 1999 | USA | Medicinal plant knowledge | 2 |
| 57 | Ohmagari and Berkes | 1997 | Canada | Craft and land skills | 4 |
| 58 | Olowa et al. | 2012 | Philippines | Medicinal plant knowledge | 1 |
| 59 | Oteros-Rozas et al. | 2013 | Spain | Agricultural and farming knowledge | 3 |
| 60 | Panghal et al. | 2010 | India | Medicinal plant knowledge | 2 |
| 61 | Patankar et al. | 2015 | Nicobar (India) | Sea knowledge | 1 |
| 62 | Pearce et al. | 2011 | Canada | Craft and land skills | 4 |
| 63 | Pilgrim et al. | 2008 | UK, India and Indonesia | Ethnobotanical knowledge | - |
| 64 | Polo et al. | 2009 | Spain | Ethnobotanical knowledge | 2 |
| 65 | Quinlan and Quinlan | 2007 | Dominica | Medicinal plant knowledge | 2 |
| 66 | Ramstad et al. | 2007 | New Zealand | Animal knowledge | 1 |
| 67 | Reyes-Garcia et al. | 2007 | Bolivia | Ethnobotanical knowledge | - |
| 68 | Reyes-Garcia, Guèze et al. | 2013 | Bolivia | Ethnobotanical knowledge | 3 |
| 69 | Reyes-Garcia, Luz et al. | 2013 | Bolivia | Animal knowledge | 3 |
| 70 | Rotherham | 2007 | UK | Agricultural and farming knowledge | 3 |
| 71 | Salick et al. | 1997 | Peru | Ethnobotanical knowledge | 3 |
| 72 | Santha | 2008 | India | Sea knowledge | 1 |
| 73 | Sayne-Vasquez et al. | 2013 | Mexico | Ethnobotanical knowledge | 3 |
| 74 | Simsek et al. | 2004 | Turkey | Ethnobotanical knowledge | 1 |
| 75 | Schunko et al. | 2012 | Austria | Ethnobotanical knowledge | 2 |
| 76 | Sogbohossou et al. | 2015 | Benin | Ethnobotanical knowledge | 3 |
| 77 | Spoon | 2011 | Nepal | Environmental knowledge | - |
| 78 | Srithi et al. | 2009 | Thailand | Medicinal plant knowledge | 3 |
| 79 | Sujarwo et al. | 2014 | Indonesia | Ethnobotanical knowledge | 3 |
| 80 | Trillo et al. | 2010 | Argentina | Medicinal plant knowledge | 2 |
| 81 | Tsuji | 1996 | Canada | Animal knowledge | 2 |
| 82 | Turner and Turner | 2008 | Canada | Ethnobotanical knowledge | 3 |
| 83 | Uprety et al. | 2012 | Nepal | Ethnobotanical knowledge | 1 |
| 84 | Vandebroek and Balick | 2012 | USA-Dominican Republic | Medicinal plant knowledge | - |
| 85 | Voeks | 2010 | Brazil | Medicinal plant knowledge | 2 |
| 86 | Voeks and Leony | 2004 | Brazil | Medicinal plant knowledge | 2 |
| 87 | von Glasenapp and Thornton | 2011 | Switzerland | Agricultural and farming knowledge | 3 |
| 88 | Wester and Yongvanit | 1995 | Thailand | Ethnobotanical knowledge | 3 |
| 89 | Yineger et al. | 2008 | Ethiopia | Medicinal plant knowledge | 2 |
| 90 | Zarger and Stepp | 2004 | Mexico | Ethnobotanical knowledge | 1 |
| 91 | Zent | 2001 | Venezuela | Ethnobotanical knowledge | 3 |
| 92 | Zobolo and Mkabela | 2006 | South Africa | Medicinal plant knowledge | 2 |
